# Whole brain radiotherapy for brain metastases from breast cancer: estimation of survival using two stratification systems

**DOI:** 10.1186/1471-2407-7-53

**Published:** 2007-03-26

**Authors:** Gustavo A Viani, Marcus S Castilho, João V Salvajoli, Antonio Cassio A Pellizzon, Paulo E Novaes, Flavio S Guimarães, Maria A Conte, Ricardo C Fogaroli

**Affiliations:** 1 Radiation Oncology Department, Hospital do Cancer, São Paulo, Brazil.

## Abstract

**Background:**

Brain metastases (BM) are the most common form of intracranial cancer. The incidence of BM seems to have increased over the past decade. Recursive partitioning analysis (RPA) of data from three Radiation Therapy Oncology Group (RTOG) trials (1200 patients) has allowed three prognostic groups to be identified. More recently a simplified stratification system that uses the evaluation of three main prognostics factors for radiosurgery in BM was developed.

**Methods:**

To analyze the overall survival rate (OS), prognostic factors affecting outcomes and to estimate the potential improvement in OS for patients with BM from breast cancer, stratified by RPA class and brain metastases score (BS-BM). From January 1996 to December 2004, 174 medical records of patients with diagnosis of BM from breast cancer, who received WBRT were analyzed. The surgery followed by WBRT was used in 15.5% of patients and 84.5% of others patients were submitted at WBRT alone; 108 patients (62.1%) received the fractionation schedule of 30 Gy in 10 fractions. Solitary BM was present in 37.9 % of patients. The prognostic factors evaluated for OS were: age, Karnofsky Performance Status (KPS), number of lesions, localization of lesions, neurosurgery, chemotherapy, absence extracranial disease, RPA class, BS-BM and radiation doses and fractionation.

**Results:**

The OS in 1, 2 and 3 years was 33.4 %, 16.7%, and 8.8 %, respectively. The RPA class analysis showed strong relation with OS (p < 0.0001). The median survival time by RPA class in months was: class I 11.7, class II 6.2 and class III 3.0. The significant prognostic factors associated with better OS were: higher KPS (p < 0.0001), neurosurgery (P < 0.0001), single metastases (p = 0.003), BS-BM (p < 0.0001), control primary tumor (p = 0.002) and absence of extracranial metastases (p = 0.001). In multivariate analysis, the factors associated positively with OS were: neurosurgery (p < 0.0001), absence of extracranial metastases (p <0.0001) and RPA class I (p < 0.0001).

**Conclusion:**

Our data suggests that patients with BM from breast cancer classified as RPA class I may be effectively treated with local resection followed by WBRT, mainly in those patients with single BM, higher KPS and cranial extra disease controlled. RPA class was shown to be the most reliable indicators of survival.

## Background

Brain metastases (BM) are the most common form of intracranial cancer. They exceed the number of primary brain tumors by at least ten times and occur in about 25% of all patients with cancer. Most brain metastases originate from lung (40–50%), breast (15–25%), melanoma (5–20%), and kidney (5–10%). Brain metastases are located in the cerebral hemispheres in about 80%, in the cerebellum in 15%, or in the brainstem in 5% of patients [1]. The median survival of untreated patients may be as short as 1–2 months [2-4]. After radiation therapy an increase in survival is reported in the range from 3 to 6 months [4-6]. The incidence of brain metastases seems to have increased over the past decade, and may be the paradoxical result of the effectiveness of drugs that do not cross the blood – brain barrier (BBB). As a result of the increased survival in patients receiving chemotherapy, brain metastases may become symptomatic [7,8]. Recursive partitioning analysis (RPA) of data from three Radiation Therapy Oncology Group (RTOG) trials (1200 patients) has allowed three prognostic groups to be identified [9]. RPA class was initially developed to categorize patients treated with fractionated external beam brain RT and tested in the radiosurgical treatment of BMs [10-12]. More recently, Lorenzoni et al. [13] proposed a simplified stratification system that uses the evaluation of three main prognostics factors for radiosurgery in brain metastases; this system was called of basic score for brain metastases (BS-BM), and may be calculated by adding the scores (0 or 1) of three main prognostic factors: KPS, control of the primary tumor, and existence of extracranial metastases, ranging from 0 (worst condition) to 3 (best condition) [13]. In this way, the intention of present study was to analyze the prognostic factors in our series of patients with brain metastases from breast cancer treated with Whole brain radiotherapy (WBRT), with an emphasis on to test the potential improvement in survival for patients stratified by the one previously described stratification system for radiosurgery (BS-BM) and compares it with the RTOG recursive partitioning analysis.

## Methods

The records of 174 patients with brain metastases, who were treated with WBRT at our institution between January 1996 and December 2004, were analyzed retrospectively. The institutional review boards granted ethical approval of the study. In the sample of the current study (n = 174) we use 81 patients with diagnoses of breast cancer who had been part of a previous study on WBRT for Brain metastases from any site [14]. The study was approved by the institutional review boards. In present study, at diagnosis of brain metastasis, the following variables were analyzed for survival: age, location of brain metastasis, extent of disease, initial Karnofsky score, dose and fractionation of radiotherapy, surgical resection, chemotherapy, RPA class and BS-BM, as showed in table-1. Chemotherapy was administered to the patients with systemic disease in activity at same time that WBRT. Supportive care (oral corticosteroids) and neurological status were not evaluated. Brain metastases were detected by contrast-enhanced cerebral computed tomography (CT) or magnetic resonance imaging (MRI). Primary tumor control was defined as remission or stable disease, without any clinical, radiologic, or laboratory findings suggestive of tumor progression at 2 months before WBRT. According to this criteria twenty patients had local or locoregional relapse, the others patients (n = 68) had brain metastases as first cancer diagnosis. WBRT was performed in all patients with cobalt 60 gamma rays or with 4 MV photons of a linear accelerator. The whole brain was irradiated by usual opposed lateral fields encompassed the cranium with a 1 cm margin. Individual shielding blocks were fabricated for all patients, when necessary. Forty two patients received WBRT with fields included leptomeninges. The total dose was 30–40 Gy, with a median of 35 Gy, in daily fractions of 2.0–3.0 Gy. During the study period two fractionation schemes were used: conventional fractionation with daily fractions of 2 Gray (Gy), five days per week to a planned total dose of 40 Gy (n = 66) and hypofractionation with daily fractions of 3 Gy, five days per week to a planned total dose of 30 Gy (n = 108). Surgical resection was indicated in single brain metastases with diameter less than or equal to 3 cm, favorable localization and controlled systemic disease. Biopsy alone and subtotal resection were done in 2 and 1 cases, respectively. All the others patients (n = 24) submitted to surgical resection were considered gross total resection. The supportive care (prednisone oral) was introduced at the beginning of treatment or during radiotherapy. The RPA was used to classify the patients with brain metastases Class I contained all patients with a Karnofsky performance status (KPS) ≥ 70, age < 65 years, controlled primary tumor and no extracerebral metastases, Class III contained patients with a KPS <70, and Class II contained all other patients [9]. BS-BM was calculated by adding the scores (0 or 1) of three main prognostic factors: KPS, control of the primary tumor, and existence of extracranial metastases [13]. The BS-BM ranged from 0 (worst condition) to 3 (best condition).

### Statistical Analysis

The endpoint of the study was overall survival. The survival time was calculated from the starting date of WBRT to the date of death or last patient contact using the method of Kaplan Meier. Survival curves were compared using the log-rank test. The covariates examined in all cases were: age, sex, location of brain metastasis, extracranial disease, control of primary tumor, initial Karnofsky score, dose and fractionation radiotherapy schedule, surgical resection, RPA class and BS-BM. All factors with a P-value ≤ 0.05 at univariate analysis were entered into a multivariate analysis using the proportional hazards model (Cox Regression) with confidential interval of 95%.

## Results

**Figure 1:**
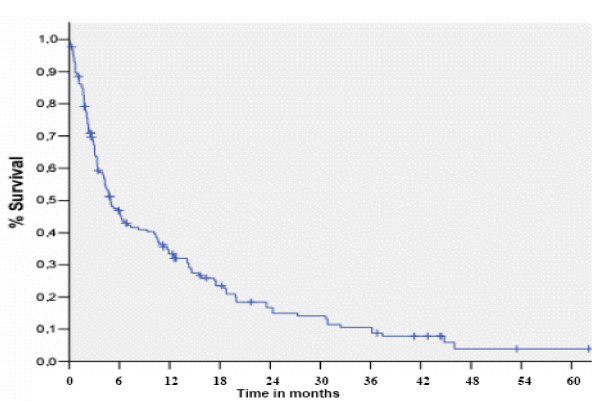
Overall Survival (Kaplan Meier estimate).

The overall survival rate in 1, 2 and 3 years was 33.4 %, 16.7%, and 8.8 %, respectively (figure-1). The interval between the diagnoses of breast cancer and brain metastasis was 32 months (range 0 – 74). The RPA class analysis showed strong relation with survival (p < 0.0001), table-2. The median survival time by RPA class in months was: class I 11.7, class II 6.2 and class III 3.0 as showed in table-3. According to BS-BM system, the median survival was of 24.6 months for patients with a score of 3, 6.6 months for patients with a score of 2, 4.7 months for patients with a score of 1, and 2.8 months for patients with a score of 0 (*p *< 0.0001), as demonstrated in table-3. Three patients were alive in moment of this analysis with a median survival time of 4.42 years (range, 3.8 – 5.1). All these patients had single brain metastasis, high KPS, cranial extra disease controlled, submitted to surgical resection before WBRT, had RPA class 1 and BS-BM 3.

**Figure 2:**
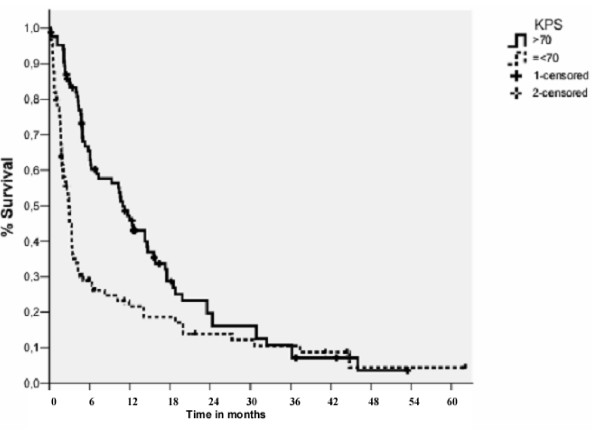
Overall Survival by KPS (Log Rank Test).

**Figure 3:**
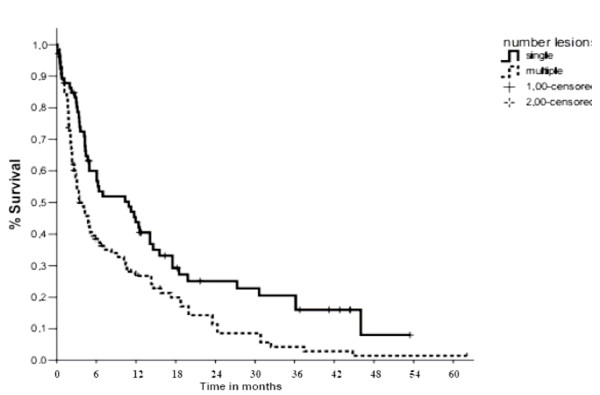
Overall Survival by Number of Lesions (Log Rank Test).

**Figure 4:**
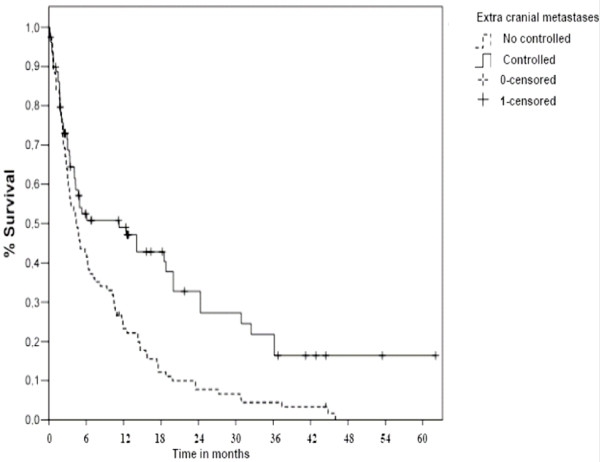
Overall Survival by Extra Cranial Metastases (Log Rank Test).

**Figure 5:**
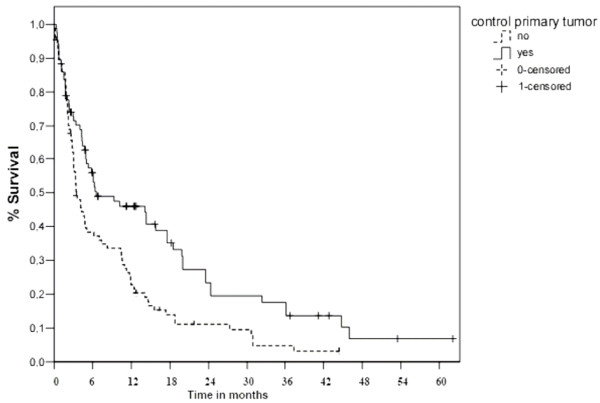
Overall Survival by Control primary tumor (Log Rank Test).

**Figure 6:**
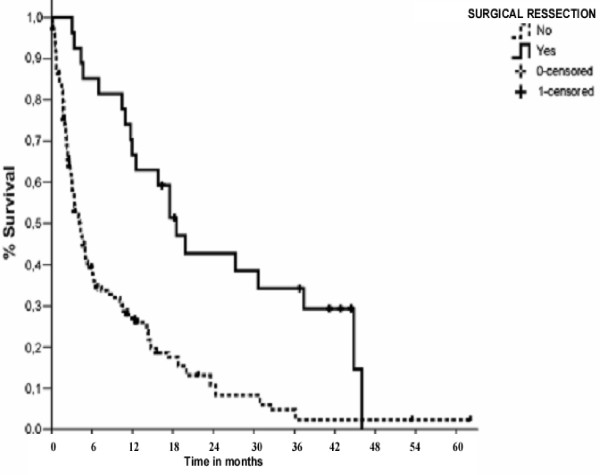
Overall Survival by Surgical Resection (Log Rank Test).

The significant prognostic factors on univariate analysis associated with better survival were: higher KPS (p < 0.0001), surgical resection (P < 0.0001), single metastases (p = 0.003), controlled primary tumor (p = 0.002) and absence of extracranial metastases (p = 0.001), as showed in table-2 and figures 2, 3, 4, 5, 6. In multivariate analysis, the factors associated positively with survival were: surgical resection (p < 0.0001), absence of extracranial metastases (p <0.0001) and RPA class I (p < 0.0001), as demonstrated in table-4.

## Discussion

Whole brain radiotherapy has traditionally been the standard treatment for patients with brain metastases since 1950. WBRT has been shown to effectively improve neurologic symptoms and function for patients with minimum co-morbidity. Breast cancer is one of the malignant tumors that frequently metastasize to the brain [17]. Once a diagnosis of brain metastasis has been established, prognosis is generally poor [14,18]. In this cohort, the overall survival rate in one year was of 33% with a median survival time 6.4 months. We previously reported a series of 270 patients with brain metastases treated with WBRT alone with or without surgical resection and, in this series, the estimated median survival was 4.6 months [14].

The table-5 contains data from the largest studies of patients with similar diseases who were followed at different institutions, and treated with different modalities. The median survival of 6.4 months reported in our study is consistent with other reports describing the natural history and treatment with WBRT alone of breast carcinoma metastatic to the brain [19,20]. Researchers reporting on different treatment modalities in similar groups of patients have noted median survival times in the range of 4 – 16 months after surgery with or without WBRT, SRS or WBRT alone [19-25], as showed in table-5.

The RTOG has evaluated a number of different radiation fractionation schemes, but median survival seems independent of the dose and schedule [26-29]. In our study, total dose of WBRT was not a statistically significant predictor of overall survival. Surgery is an important modality for patients with a single brain metastasis, particularly when favorable prognostic factors and systemic disease control are present [30,31]. Our data showed that patients undergoing resection of brain lesion followed by WBRT was associated with significantly better overall survival (p < 0.0001) than patients submitted to biopsy or WBRT alone. Patchell et al [15], randomly assigned 48 patients with single brain metastases (10% with breast primaries) to surgery followed by WBRT versus WBRT alone. Patients in the combined arm experienced a longer duration of functional independence (38 v 8 weeks), and improved survival (40 v 15 weeks; P < .01). Noordijk et al [30] conducted a randomized trial of 63 patients (19% with breast primaries) that confirmed and extended these findings. Importantly, in this study, the benefit of combined-modality therapy was seen only in patients with stable or absent extracranial disease. Patients with progressive extracranial disease at study entry achieved a median survival of only 5 months, irrespective of the allocated treatment. One additional trial failed to demonstrate a survival or quality-of-life benefit [31]. Nearly half of the patients in this trial had extracranial disease, and 10 of 43 patients randomly assigned to radiotherapy underwent surgical resection.

The end point of this cohort was to evaluate the different prognostic factors related with overall survival and to analyze the importance of recursive partitioning analysis (RPA) class (RTOG) in patients with brain metastasis. In our data, the prognostic factors in the univariate analysis associated with better survival were: Higher KPS, solitary metastasis, surgical resection, RPA class I, BS-BM -3, control primary tumor, and absence of extracranial metastases; in the multivariate analysis RPA class I and surgical resection maintained associated with better survival, being all these prognostic factors were showed for others authors in previous studies [9,14,16].

In recent publication, the Radiation Enhancing Allosteric Compound for Hypoxic Brain Metastases (REACH) study [32] tested the hypothesis that adding efaproxiral to WBRT plus supplemental oxygen would improve survival better than WBRT with supplemental oxygen alone. The results of this study suggest that efaproxiral, may improve response rates to WBRT and survival in patients with brain metastases, mainly metastases from breast cancer. Moreover, in this phase III study; KPS, number of extracranial metastatic sites, and sex had the highest statistical significance in multivariate analysis. In our study, the others factors (age, chemotherapy, dose and fractionation schedule) analyzed were not associated with any effect in survival. RPA class in this study showed similar results to RTOG protocols to identify patients with different results [9], with the median survival time for class I (11.5 months), II (6.2 months) and III (3.0 months) (p = 0.0001), respectively. In this series, the BS-BM was effective in identifying patients with different outcomes in a simple and easy manner. A BS-BM of 0 had greater specificity but lower sensitivity BS-BM. However, in our study BS-BM when compared to RPA class in multivariate analyses did not achieved significant statistical in Cox regression backward method, this data shows that RPA class is more powerful and precise than BS-BM in to predict survival for patients with brain metastases from breast cancer. Thus, theses results do not invalidated its use as a system for predict survival, only it confirms that the RPA is a more efficient system for this. But, which was the reason for this to occur? Probably this fact occurred because the BS-BM takes into account only three variables (i.e., KPS, primary tumor control, and the presence of extracranial metastases), which have been found in most studies, as well as in our own evaluation, to be the most important prognostic factors for survival. Thus it seems that less important factors had been affected indirectly by the other main factor as extracranial metastases or surgical resection of lesions. Patients with three or more BMs had a greater proportion of extracranial metastases and smaller than surgical resection of lesions than those with one or two BMs (48%vs 22 % and 26% vs 58 %, respectively). In this way, our data showed that BS-BM system may be used effectively in patients with brain metastases treated by WBRT alone or combined with surgery.

## Conclusion

In conclusion, both the stratification systems examined were able to identify quite well those patients who might or might not benefit from WBRT. RPA class was shown to be the most reliable indicators of survival. BS-BM has the advantage of focusing on only three major factors for survival. Our data suggest that patients with brain metastases from breast cancer classified as RPA class I may be effectively treated with local resection followed by WBRT, mainly in those patients with single metastases, higher KPS and cranial extra disease controlled. We believe that patients presenting with a RPA Class III or BS-BM of 0 are clearly not good candidates for surgical resection followed by WBRT. Patients with RPA Class II or BS-BM of 1 in general have a poor outcome, and, in these patients, the decision concerning treatment remains difficult. Despite the generally ominous prognosis, some patients still benefit from surgical ressection. Brain metastases from breast cancer pose numerous challenges. Future areas of research may include characterization of risk factors and in this way to evaluated new approaches for the treatment of brain metastases.

## Competing interests

The author(s) declare that they have no competing interests.

## Authors' contributions

VG carried out the patient recruitment, acquisition and interpretation of the data. He also drafted the manuscript. VG performed the statistical analysis and drafted the manuscript. CM participated in the design of the study, carried out the patient recruitment and gave final approval of the version to be published. S JV and G FS participated in the design of the study, P AC carried out the patient recruitment and gave final approval of the version to be published, N PE carried out the patient recruitment and gave final approval of the version to be published, F R participated in the design of the study and C MA gave final approval of the version to be published. All authors read and approved the final manuscript.

## Pre-publication history

The pre-publication history for this paper can be accessed here:



## Figures and Tables

**Table 1:** Characteristic of treatment and patients

***AGE***	***MEDIAN***	***RANGE***
Patients	57	38 – 82
***BRAIN METASTASES SCORE***	***NUMBER***	***%***
**BM-S O**	34	19.5
**B*M-S 1***	54	31
**BM-S 2**	61	35
**BM-S 3**	25	14.5
***KPS***		
< 70	89	51.1
>= 70	85	48.8
***SURGICAL RESECTION***		
Yes	27	15.5
No	147	84.5
***DOSE(Gy)*** ***FRACTIONATION (fr)***		
40 Gy/20 fr	66	37.9
30 Gy/10 fr	108	62.1
***NUMBER LESIONS***		
Single	66	37.9
Multiple	108	62.1
***CHEMOTHERAPY***		
Yes	32	18.3
No	142	81.6
***RPA CLASS***		
Class I	39	22.4
Class II	46	26.4
Class III	89	51.2
***LOCALIZATION***		
Supratentorial	140	80.5
Infratentorial	24	13.8
Both	10	5.7
***PRIMARY DISEASE CONTROL***		
Yes	86	49.4
No	88	50.6
***EXTRACRANIAL METASTASES***		
Controlled	80	46
No Controlled	94	54

**Table 2:** Univariate analyses for survival (Log Rank test)

**Variable**	**Number**	**Event (n)**	**%**	**P**
AGE	132	106	OS 12 months	0.16
< 65 Years	42	36	34.8	
>= 65 Years			29.3	
KPS				<0.0001
< 70	89	75	21.7	
>= 70	85	67	45.8	
SURGICAL RESECTION				<0.0001
Yes	27	20	66.7	
No	147	122	26.9	
DOSE(Gy) FRACTIONATION (fr)				**0.11**
40 Gy/20 fr	66	55	27.6	
30 Gy/10 fr	108	87		
NUMBER LESIONS				0.003
Single	66	51	43.8	
Multiple	108	91		
CHEMOTHERAPY				0.29
Yes	32	27	47.8	
No	142	122	28.4	
RPA CLASS				< 0.0001
Class I	39	28	55.6	
Class II	46	39	37.7	
Class III	89	75	21.7	
PRIMARY DISEASE CONTROL				0.002
Yes	86	62	46	
No	88	80	22.8	
EXTRA CRANIAL METASTASIS				30.001
Yes	80	50	23.	
No	94	92	49	
BS-BM				< 0.0001
Score 0	34	33	11.8	
Score 1	54	52	24.1	
Score 2	61	44	42.6	
Score 3	25	13	69.2	

**Table 3:** Median survival according to RPA class and BS-BM score.

Characteristics* KPS >= 70, age <65 y, controlled primary no extracranial disease	RPA class I		Median survival (mo) RPA 11.7
All others	RPA class II		6.2
KPS < 70	RPA class III		3.0
BS-BM	YES OR NO		Median survival (mo) BS-BM
Primary controlled	1	0	
KPS > 70	1	0	
No metastases extra cranial	1	0	
SCORE 3	three factors present		24.6
2	two factors present		6.6
1	one factor present		4.7
0	none factor present		2.8

**Table 4:** Multivariate analyses of significant factors associated with survival by Cox regression

**VARIABLE**	**P**	**HR**	**95% confidential interval**	
**SURGICAL RESECTION**	<0.0001	4.34	2.5	7.14
**SINGLE METASTASES**	0.14	1.08	0.97	1.21
***KPS >= 70***	0.55	1.31	0.55	3.23
**BS-BM**	0.58	0.63	0.12	3.29
**RPA**	<0.0001	1.64	1.32	2.04
**CONTROL PRIMARY TUMOR**	0.66	0.92	0.63	1.34
**NO EXTRACRANIAL METASTASES**	<0.0001	2.38	1.63	3.44

**Table 5:** Outcomes of the brain metastases from breast cancer treatment according to treatment modality, number of patients, median survival and prognostic factors by other researches.

***Authors***	***Patients(n)***	***Treatment***	***Median OS(mo)***	***Prognostic factors (univarate analysis)***
**Firlik et al. (21),2000 **	58	SRS	13	---
**Pieper et al.(22),1997 **	63	Surgery+- WBRT	16	Age, menopausal status, postoperative RT, KPS, systemic disease
**Wronski et al. (23), 1996.**	70	Surgery+- WBRT	14	Positive estrogen receptor, meningeal carcinomatosis
**Lentzsch et al. (24), 1999 **	145	WBRT	6.1	KPS, dose of RT, No. of BM, grade of primary
**Mizutani et al. (19), 2001 **	113	WBRT	4.0	KPS, No. of BM, systemic metastases without bony metastasis
**Fokstuen et al. (20), 2000 **	99	WBRT	5.0	No. of BM, systemic metastases
**Mahmoud-Ahmed et al. (25) 2002**	116	WBRT	4.1	KPS, WBRT dose, RPA class
**Present study. 2006**	174	Surgery +WBRT or WBRT	6.4	KPS, RPA, single BM, resection of lesion, extracranial metastase, control primary tumor, BS-BM system
